# Breakpoint drift: a hidden confounder in antimicrobial resistance surveillance?

**DOI:** 10.3389/fmicb.2025.1674968

**Published:** 2025-08-29

**Authors:** Malik Sallam

**Affiliations:** ^1^Department of Pathology, Microbiology and Forensic Medicine, School of Medicine, The University of Jordan, Amman, Jordan; ^2^Department of Clinical Laboratories and Forensic Medicine, Jordan University Hospital, Amman, Jordan

**Keywords:** guideline adherence, microbial sensitivity tests, drug resistance, AMR (antimicrobial resistance), AST (antibiotic susceptibility testing)

## 1 Introduction

It is a well-established biological principle that exposure to antimicrobials imposes selective pressure on microbial populations (Charlebois, 2023; Hasan et al., 2021). This selective pressure favors the survival and propagation of resistant microbial sub-populations (Cantón and Morosini, 2011). The emergence of resistance under such conditions is both expected and extensively documented, supported by empirical evidence, mechanistic studies, and molecular analyses of genetic adaptation (Holmes et al., 2016; Muteeb et al., 2023). Accordingly, the phenomenon itself is not in question. Rather, it is the methodological framework through which resistance is defined and monitored that warrants critical examination, particularly in light of continued breakpoint changes.

A potentially consequential concern lies not in the biology of the organism itself, but in the interpretive frameworks through which laboratory data are classified. The widely accepted narrative of rising antimicrobial resistance (AMR) may reflect not only genuine microbial evolution, but also the cumulative impact of evolving interpretive standards—particularly revisions to minimum inhibitory concentration (MIC) breakpoints and zone diameter thresholds that determine categorical susceptibility (Hombach et al., 2012). These redefinitions, though grounded in scientific rationale, raise the possibility that some observed increases in AMR may result from shifting standards rather than true biologic changes of the tested infectious agents.

## 2 Breakpoint drift and its implications

In clinical microbiology, there is a foundational trust in the objectivity of diagnostic tools—the defined inhibition zone diameters, the broth microdilution assays that quantify minimum inhibitory concentrations (MICs), and the interpretive algorithms that generate categorical antimicrobial susceptibility testing (AST) results (Kowalska-Krochmal and Dudek-Wicher, 2021). Tools such as the Advanced Expert System (AES) further integrate these outputs into therapeutic recommendations (Winstanley and Courvalin, 2011). However, these tools produce raw data, not clinical meaning. Interpretation is contingent on breakpoints established by standards-setting organizations such as the Clinical and Laboratory Standards Institute (CLSI) and the European Committee on Antimicrobial Susceptibility Testing (EUCAST) (Gaur et al., 2023). These interpretive thresholds evolve in response to emerging pharmacokinetic/pharmacodynamic (PK/PD), microbiologic, and clinical outcome data. Although such revisions are scientifically justified, they have significant implications for how AMR is defined, reported, and interpreted over time (Humphries et al., 2019).

The systematic analysis conducted by Hombach et al. (2012)provided compelling evidence of how shifts in interpretive breakpoints can significantly alter reported AMR rates—independent of any underlying biological change. The study demonstrated that applying updated CLSI and EUCAST breakpoints to a large collection of Gram-negative isolates led to substantial increases in reported resistance rates—solely due to changes in interpretive criteria (Hombach et al., 2012). Resistance classifications for several key pathogens and antimicrobial classes shifted significantly, despite no change in the underlying microbiology. These findings highlight how revised breakpoints alone can alter perceived resistance patterns, emphasizing the importance of accounting for such changes in longitudinal surveillance and trend analyses (Hombach et al., 2012).

Such changes in interpretive criteria constitute more than technical revisions—they represent fundamental shifts in the conceptual framework through which AMR is defined. For example, a bacterial isolate with an inhibition zone diameter previously categorized as susceptible under earlier guidelines may now be reported as resistant according to current standards—not because of recent adaptive change in the organism, but due to updated criteria that better reflect PK/PD evidence, patient factors, and contemporary clinical practice (Cardoso et al., 2025; Sader et al., 2023). Such reclassifications—illustrated by selected examples in Table 1—while improving the clinical accuracy of current susceptibility assessments, can nonetheless produce substantial shifts in reported AMR rates within surveillance datasets, cumulative antibiograms, and public health reports. Without explicit adjustment, these changes risk being misinterpreted as evidence of accelerated microbial evolution or dissemination, when in fact part of the observed change may arise from methodological redefinition rather than from changes in the underlying biology (GBD 2021 Antimicrobial Resistance Collaborators, 2024).

**Table 1:** Comparison of clinical and laboratory standards institute (CLSI) disk diffusion interpretive criteria (zone diameter, mm) for selected organism–antimicrobial combinations in 2015 and 2025.

**Bacteria**	**Antimicrobial**	**CLSI-2015 (S) ≥**	**CLSI-2025 (S) ≥**	**CLSI-2015 (I)**	**CLSI-2025 (I)**	**CLSI-2015 (R) ≤ **	**CLSI-2025 (R) ≤ **
*Acinetobacter* spp.	Ampicillin-sulbactam	15	22	12–14	17–21	11	16
*Acinetobacter* spp.	Minocycline	16	22	13–15	18–21	12	17
*Enterobacterales*	Gentamicin	15	18	13–14	15–17	12	14
*Enterobacterales*	Amikacin	17	20	15–16	17–19	14	16
*Enterobacterales*	Ciprofloxacin	21	26	16–20	22–25	15	21
*Enterobacterales*	Levofloxacin	17	21	14–16	17–20	13	16
*Pseudomonas aeruginosa*	Piperacillin-tazobactam	21	22	15–20	18–21	14	17
*Pseudomonas aeruginosa*	Ciprofloxacin	21	25	16–20	19–24	15	18
*Staphylococcus aureus* including MRSA	Ceftaroline	24	25	21–23	20–24 (SDD)	20	19
All *Staphylococci*	Linezolid	21	26	–	23–25	20	22

Values indicate the minimum inhibitory zone diameter [nearest whole millimeter (mm)] defining Susceptible (S), the Intermediate or Susceptible, Increased Exposure (I/SDD) range, and the maximum diameter defining Resistant (R), as published in CLSI Performance Standards for Antimicrobial Susceptibility Testing (2015 vs. 2025 editions) cited in Clinical and Laboratory Standards Institute (2015, 2025).

MRSA, Methicillin-resistant Staphylococcus aureus.

## 3 Discussion, recommendations, and conclusion

Currently, there is no standardized method for harmonizing historical AST data with revised clinical breakpoints, nor is there a routine acknowledgment that increases in reported resistance may, at least in part, reflect methodological reclassification rather than true microbiological change. This creates an epistemological conflation: a biological phenomenon confounded by a shifting interpretive framework. The situation is akin to changing diagnostic thresholds in chronic disease classification—for example, redefining the blood pressure cutoff for hypertension inevitably increases disease prevalence, not because more individuals have developed pathology, but because the diagnostic criteria have shifted. Similarly, AMR may appear to rise, not necessarily due to increased pathogen resilience, but because the benchmarks used to define AMR have been made more stringent.

Failure to account for evolving interpretive criteria in the analysis of AMR trends carries direct implications for public health policy and clinical decision-making. Revisions to clinical breakpoints, even when grounded in robust PK/PD and clinical evidence, function as recalibrators of AMR rates; an effect acknowledged in some studies but not consistently adjusted for in surveillance analyses (GBD 2021 Antimicrobial Resistance Collaborators, 2024). The updated CLSI breakpoints for fluoroquinolones and cephalosporins in *Enterobacterales*, as well as EUCAST's redefinition of the “intermediate” category as “susceptible, increased exposure,” are emblematic of such changes (Nabal Díaz et al., 2022; Van et al., 2019). Such changes have substantial epidemiological consequences that are seldom integrated with appropriate granularity into AMR surveillance datasets.

Public discourse, meanwhile, has been saturated with urgent headlines—“Resistance is Rising,” “Superbugs on the March”—that often conflate microbial evolution with shifts in regulatory definitions (Arias and Murray, 2009; Capurro, 2020; Painuli et al., 2023). This is not a criticism of CLSI or EUCAST, whose breakpoint revisions are evidence-based and reflect scientific progress. However, for that progress to be meaningful, its evolution must be acknowledged. Unadjusted AMR data may lead to unnecessary broad-spectrum antibiotic use, misdirected investments, and flawed policy decisions. One contributing mechanism is that such data can make certain first-line agents appear less reliable for empirical therapy, prompting earlier escalation to agents such as carbapenems in place of β-lactam/β-lactamase inhibitor combinations (Lau et al., 2022). This perception, often shaped by institutional antibiograms and surveillance reports, while well-intentioned, can increase selection pressure on last-line drugs and accelerate the emergence of multidrug-resistant organisms (Zilberberg et al., 2017). Without accounting for shifting definitions, long-term AMR trends may capture changes in classification criteria rather than true microbial evolution, risking misinterpretation of resistance dynamics and potentially misleading clinical, epidemiological, and policy decisions.

What, then, are the necessary steps forward? First, AMR surveillance—whether global, national, or institutional—should routinely annotate their reports with breakpoint metadata. Just as genome browsers present annotation tracks alongside nucleotide sequences, and as widely circulated COVID-19 incidence and mortality plots annotated changes in diagnostic definitions or reporting criteria, so too should antibiograms and AMR trend analyses display the interpretive context used at the time of data acquisition, including the version and source of the breakpoints applied. Second, academic and regulatory bodies should establish a standardized nomenclature for breakpoint epochs. Resistance data labeled as “S[CLSI2012]” or “R[EUCAST2021],” for example, would clearly communicate the interpretive framework, facilitating both cross-sectional comparison and accurate longitudinal analysis. Third, when historical bacterial isolates are available, they should be retested using contemporary breakpoints to enable true retrospective trend evaluation. Although labor-intensive, this is feasible in reference laboratories and academic centers with bio-banked microbial strains. In cases where isolates are no longer available, modeling approaches should be employed to retrospectively adjust historical data based on known MIC distributions and classification changes. Fourth, modeling consortia—such as those behind the Global Burden of Disease (GBD) project—should incorporate breakpoint harmonization protocols into their methodologies. Adjustment factors or, at minimum, sensitivity analyses are essential to account for interpretive changes across time.

In the discourse surrounding AMR, a fundamental question must be asked: does our current narrative reflect true microbial evolution, or the cumulative effect of evolving diagnostic conventions? Increases in AMR prevalence may reflect not only bacterial adaptation but also redefinition through processes such as breakpoint drift; neglecting the latter risks incomplete or biased interpretation of surveillance data. The truth, as always, lies at the intersection of both. Microorganisms evolve, undoubtedly—but so too does the diagnostic lens through which we measure that evolution. Recognizing the profound implications of breakpoint recalibration and the confounding effects of breakpoint drift should not diminish the urgency of addressing AMR; rather, it should strengthen the foundation upon which our understanding rests. To address AMR effectively as a major medical challenge of the 21st century, we must ensure that our measurement tools keep pace with both microbial evolution and advances in diagnostic methodology, so that changes in reported resistance reflect true shifts in biology rather than artifacts of classification.
